# Prediction of eye color in the Slovenian population using the IrisPlex SNPs

**DOI:** 10.3325/cmj.2013.54.381

**Published:** 2013-08

**Authors:** Vanja Kastelic, Ewelina Pośpiech, Jolanta Draus-Barini, Wojciech Branicki, Katja Drobnič

**Affiliations:** 1 National Forensic Laboratory, General Police Directorate, Police, Ministry of the Interior, Ljubljana, Slovenia; 2 Institute of Forensic Research, Section of Forensic Genetics, Kraków, Poland; 3 Department of Genetics and Evolution, Institute of Zoology, Faculty of Biology and Earth, Kraków, Poland

## Abstract

**Aim:**

To evaluate the accuracy of eye color prediction based on six IrisPlex single nucleotide polymorphisms (SNP) in a Slovenian population sample.

**Methods:**

Six IrisPlex predictor SNPs (*HERC2* – rs12913832, *OCA2* – rs1800407, *SLC45A2* – rs16891982 and *TYR* – rs1393350, *SLC24A4* – rs12896399, and *IRF4* – rs12203592) of 105 individuals were analyzed using single base extension approach and SNaPshot chemistry. The IrisPlex multinomial regression prediction model was used to infer eye color probabilities. The accuracy of the IrisPlex was assessed through the calculation of sensitivity, specificity, positive predictive value (PPV), negative predictive value (NPV), and the area under the receiver characteristic operating curves (AUC).

**Results:**

Blue eye color was observed in 44.7%, brown in 29.6%, and intermediate in 25.7% participants. Prediction accuracy expressed by the AUC was 0.966 for blue, 0.913 for brown, and 0.796 for intermediate eye color. Sensitivity was 93.6% for blue, 58.1% for brown, and 0% for intermediate eye color. Specificity was 93.1% for blue, 89.2% for brown, and 100% for intermediate eye color. PPV was 91.7% for blue and 69.2% for brown color. NPV was 94.7% for blue and 83.5% for brown eye color. These values indicate prediction accuracy comparable to that established in other studies.

**Conclusion:**

Blue and brown eye color can be reliably predicted from DNA samples using only six polymorphisms, while intermediate eye color defies prediction, indicating that more research is needed to genetically predict the whole variation of eye color in humans.

Prediction of human visible characteristics by genotyping informative polymorphisms in DNA opens up a new perspective in the forensic field. Multiple genes including *HERC2, OCA2, MC1R, SLC24A5, SLC45A2, TYR, TYRP1, ASIP, SLC24A4, TPCN2, KITLG*, and *IRF4* have been associated with eye, hair, and skin color in European populations and they have been used in studies dealing with eye color prediction (1-14). Variation of iris color depends on the content of eumelanine, a brown light-absorbing biopolymer, which is present in higher concentrations in brown-eyed individuals (15,16). Although eye color is evidently a continuous variable, it has been often classified into three categories – blue, brown, and intermediate (4,14). Eye color variability is particularly striking in European populations, constituting a highly differentiating trait of potential use in forensic investigations (7,14,17). Recent studies have shown that a significant fraction of human iris color variation can be explained by polymorphisms within a single region in the human genome, comprising the evolutionary conserved *HERC2* gene and the neighboring *OCA2* gene located on the chromosome 15. It is assumed that the level of expression of the known pigmentation gene – *OCA2* – is controlled by polymorphism rs12913832 on *HERC2* locus (18,19). The remaining genes that have been shown to contribute to eye color variation are *SLC24A4, SLC45A2, TYR,* and *IRF4* (4,20,21). However, their impact on eye color prediction is lower and it seems to vary between populations (8,14,22,23). Since such differences may potentially affect accuracy of prediction in various populations, we further addressed this issue and analyzed a population sample of individuals with defined eye color from Slovenia.

Several prediction models have already been proposed to be useful in eye color prediction (4,8,9,17,23,24). Here we used six IrisPlex predictors, which were selected by Liu et al (4) from a larger set of polymorphisms potentially influencing pigmentation in humans and included into the IrisPlex prediction system (4,13,17). The IrisPlex prediction model is based on a multinomial logistic regression method and uses phenotype and genotype data from 3804 Dutch individuals. Based on these data the model gives three probabilities for blue, brown, and intermediate eye color (13). From the obtained probabilities, the most probable iris color is predicted based on recommendations given in Walsh et al (13).

## Material and methods

### Sample collection, DNA extraction, and quantification

The study population comprised 105 unrelated Slovenian volunteers, 70 male and 35 female, who signed a written consent for their DNA to be used in the project. The study was approved by the National Medical Ethics Committee of the Republic of Slovenia. The eye color was defined according to descriptions provided by the volunteers and our own grading. For confirmation and in order to prevent bias, photographs of each donor’s eyes were taken. Participants were divided into three categories according to eye color: blue, intermediate, and brown. The intermediate group included individuals with green eyes (lighter phenotype), hazel eyes (darker phenotype), and with combination of two or more pigments within the iris, such as blue or green eye color with brown peripupillary rings. The blue and the brown group included the individuals with the eye color that was clearly composed of only one color including all the shades of this particular color. Buccal swabs were collected from all volunteers using a SAFE^®^ Box kit (ForensiX, Prionics AG, Zurich, Switzerland). DNA was extracted from the samples using Chelex extraction (25). DNA extracts were quantified using the Quantifiler Human DNA Quantification Kit (Applied Biosystems Inc., Foster City, CA, USA) in accordance with the manufacturer's guidelines.

### Single nucleotide polymorphisms (SNP) genotyping

Four SNPs (*HERC2* – rs12913832, *OCA2* – rs1800407, *SLC45A2* – rs16891982, and *TYR* – rs1393350) were genotyped previously as described in Kastelic et al (26). The remaining two IrisPlex SNPs (*SLC24A4* – rs12896399 and *IRF4* – rs12203592) were genotyped for the purpose of this study using the protocol described by Walsh et al (17). Marker details and primer sequences are listed in Supplementary Tables 1 and 2(web extra material 1). All cleaned products were analyzed on the ABI Prism 3130 Genetic Analyzer (Applied Biosystems) using run parameters as described previously (17,26).

**Table 1:** Parameters describing the accuracy of prediction with the IrisPlex model*

	Color		
Parameter	blue	intermediate	brown
Area under the receiver operating characteristic curve	0.966	0.796	0.913
Sensitivity (%)	93.6	0	58.1
Specificity (%)	93.1	100.0	89.2
Positive predictive value (%)	91.7	x*	69.2
Negative predictive value (%)	94.7	74.3	83.5

*x – zero in denominator.

### Model-based prediction of eye color and evaluation of its accuracy

On the basis of the formula provided by Liu et al (4) and implemented in the eye color prediction model of the IrisPlex system, three prediction probability values were generated for each of the three phenotype categories (blue, intermediate, and brown) (Supplementary Table 3(Supplementary Table 3)). The overall prediction accuracy was assessed as previously explained by calculating area under the curve (AUC) values using SPSS 19.0 (SPSS Inc., Chicago, IL, USA) (26). The AUC is the integral of receiver operating characteristic (ROC) curve, and ranges from 0.5, which represents a total absence of prediction, to 1.0, which represents a perfect prediction. Additionally the values of sensitivity, specificity, positive predictive value (PPV), and negative predictive value (NPV) were calculated according to Liu et al using prediction threshold at the ≥0.7 level, which has been determined to be the most appropriate (1). Inconclusive results (below the threshold 0.7) were considered as negative results. From these, false negatives were used to calculate sensitivity and true negatives were used to calculate specificity.

## Results

### Characteristics of the study population

The frequency of blue eye color in the studied sample was 44.7% (47 samples) and the frequency of brown eye color was much lower and reached 29.6% (31 samples). The individuals were categorized in these two eye color groups only when the color was homogenous, regardless of the intensity. The frequency of individuals in the intermediate eye color group was relatively high, 25.7% (27 samples).

### Prediction accuracy of the IrisPlex model

**Figure 1:**
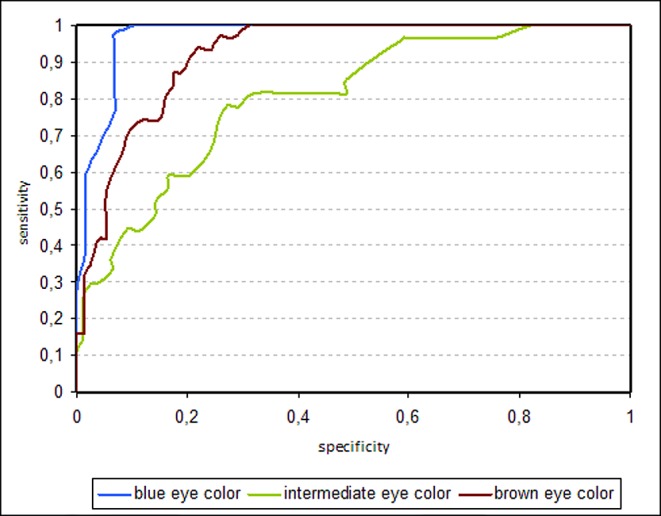
Receiver operating characteristic curve analysis of 105 Slovenian samples based on the IrisPlex prediction model.

Prediction accuracy expressed by the AUC (Figure 1) was 0.966 for blue, 0.913 for brown, and 0.796 for intermediate eye color (Table 1). The highest sensitivity was obtained for blue eye color and reached 93.6%, which means that 93.6% (44/47 individuals) of the analyzed blue-eyed persons were predicted correctly. The sensitivity for brown eye color was lower and amounted to 58.1% (18/31 individuals). The specificity values for blue (93.1%) and brown (89.2%) eye colors were very high. This means that 93.1% of non-blue individuals and 89.2% of non-brown individuals were correctly recognized as non-blue and non-brown, respectively. The highest PPV was obtained for blue eye color at the 91.7% level. This value means that in all cases of assignments to blue eye color category, 91.7% individuals in fact had blue eye color. The PPV value for brown eye color was lower and reached 69.2% and the PPV value for intermediate eye color could not be determined due to the fact that in no cases the intermediate eye color was predicted as intermediate. The NPV was very high for these two eye color categories. For blue eye color, NPV was 94.7% and this means that out of all cases where individuals were classified to the non-blue-eyed category, 94.7% cases were correctly classified as non-blue-eyed individuals. NPV for brown eye color equaled 83.5% and for intermediate eye color 74.3% (Supplementary Figure(Supplementary Figure)). A complete lack of sensitivity (0%) was observed for intermediate eye colors. This means that out of the 27 samples tested, none was classified as intermediate. Four out of 27 intermediate samples were categorized as blue with very high probabilities (>0.9). Eight (probability values above 0.7) and as many as 14 (probability values above 0.5) out of 27 intermediate samples were categorized as brown (Supplementary Figure).

## Discussion

In the studied population sample, blue eye color was present with the frequency 44.7%, while according to the Eupedia (*http://www.eupedia.com/europe/maps_of_europe.shtml#eye_colour*), the expected frequency of light eyed individuals in Slovenia should be between 50% and 79%. However, most of the individuals included in the intermediate category had green irises and blue irises with brown spots or peripupillary rings and therefore could be included in the group of light eyed people. Taking this into account, it can be said that the percentage of light-eyed individuals in the study was 56%, which is in accordance with the Eupedia.

The IrisPlex system includes six SNPs located on six genes (*HERC2* rs12913832, *OCA2* rs1800407, *SLC24A4* rs12896399, *SLC45A2* rs16891982, *TYR* rs1393350, and *IRF4* rs12203592), which are considered to be major genetic predictors of eye color (4,12-14). Numerous studies have confirmed that rs12913832 located on *HERC2* gene alone carries most of the eye color predictive information and is therefore the best known eye color predictor (4,5,8,14,22,27). We confirmed these results and also provided evidence that the CC genotype on rs12913832 was strongly associated with blue eye color (8,14,22,27). Among 49 Slovenian individuals carrying the CC genotype, 45 (91.8%) had blue eye color, which is comparable to other reports (93.4%) (14). In the remaining four cases, green eye color was observed and accordingly our study suggests that the CC genotype reliably predicts light eye colors. On the other hand, 12 (80%) out of 15 individuals carrying the TT genotype had brown eye color. It is worth noting that this proportion was higher (98%) in the larger EUREYE study (14). This difference may be a consequence of a relatively small sample set in this study, especially in brown eye color category, but also of differences in phenotype description among various studies. The latter is supported by phenotype distribution among CT genotype carriers – only 19 (46.3%) out of 41 individuals with the CT genotype had brown eyes in our population compared to 75.5% in Walsh et al (14). However, among the remaining 22 individuals, 2 had blue eye color but with intense brown peripupillary rings, 2 had green eye color, and 18 had hazel eye color. Overall, of the 56 individuals carrying the T allele either as homozygote or heterozygote state, 31 (55.4%) had brown eye color and even 49 (87.5%) had dark irises (brown or hazel). Notably, 8 (53.3%) individuals out of the 15 with a TT genotype had dark brown eye color and 4 (26.7%) had lighter brown eye color, which implies that other polymorphisms are important modifiers of eye color intensity. This study confirmed the prevailing role of the rs12913832 in the determination of blue and brown eye color. The significance of this position for human pigmentation seems to be undisputable and has been confirmed also by functional genome analysis (19). This huge effect on eye color detected in all the so far studied populations makes this SNP a key element of all eye color prediction methods. Moreover, it has been shown that this position also has influence on variation in hair and skin color (1,22). Indeed, rs12913832 is also an important element of hair color prediction models (1,3,17).

*OCA2* SNP rs1800407, which ranked second among the best eye color predictors, has very low frequency of allele A (11.9%) and therefore may have had weak overall influence on variation in eye color in our population sample (4,12,13). The remaining IrisPlex predictors have been shown to have smaller effect on iris color variation but all six are implemented in the IrisPlex macro (13). It has been pointed out that IrisPlex can accurately predict blue and brown eye color while it is inefficient in the prediction of intermediate eye color and thus one should expect considerably lower prediction accuracy for this eye color category (Figure 1) (14). Indeed, the AUC values for blue and brown eye color categories in the Slovenian population were found to be very high and equaled 0.966 and 0.913, respectively. This result is similar to the AUC values obtained using multinomial logistic regression for a much larger group of seven European populations, which were 0.964 for blue and 0.956 for brown (14). Mengel-From et al (5) investigated *HERC2, OCA2,* and *SLC45A2* variation in 395 Danes using logistic regression and concluded that variation in *HERC2-OCA2* complex can be useful for reliable prediction of light and dark eye colors (5). Pośpiech et al (7) used Bayesian network built on 638 Poles and by testing 80 samples obtained AUC values of 0.783 and 0.583 for blue and brown color, respectively (7). These values were calculated from the scoring results rather than from probabilistic values, which is a much more conservative approach. They confirmed high sensitivity of prediction of blue eye color (80%) and lower for brown eye color (35%). They also concluded that eye color can be reliably predicted from the available DNA markers at the level light-dark, obtaining a high AUC value of 0.925 (7). A similar AUC value for light and dark eye color categories (AUC = 0.985) was obtained in our previous study involving the same Slovenian population and a different set of predictors (26). The accuracy values were also similar in the study of six European populations (with AUC values 0.986 and 0.978 for blue and brown, respectively) (8). In this latter work, a different model building data set was used, which could certainly influence the final AUC values. Recently, Allwood and Harbison (23) proposed a novel eye color prediction method utilizing classification tree approach and predicted blue and brown eye color with very high accuracy (23). Notably, all the mentioned studies reported serious difficulties with prediction of intermediate eye colors, indicating that regardless of the prediction method used (multinomial logistic regression, likelihood ratio, classification trees) accuracy parameters for intermediate eye color were very weak. This strongly suggests that the currently available eye color predictors are insufficient to reliably predict intermediate eye colors. In our study, the intermediate category was relatively large, comprising 27 of samples (25.7%), and a complete lack of sensitivity observed in this category confirms the previous results indicating that more information is needed in order to predict intermediate phenotypes of iris color.

It is worth to mention that prediction of hair color from DNA is even more difficult. Branicki et al (1) and Walsh et al (17) both confirmed reliable prediction of red hair color, but prediction of other hair color categories is still affected by a relatively high error rate. Many problems with association studies aiming to find reliable predictors of various characteristics are caused by the continuous nature of the studied externally visible traits and the fact that loci with minor or weak effect on the phenotype are particularly difficult to discover in association studies. Therefore, there are different suggestions on phenotype description and proper eye color classification. For example Liu et al proposed a new digital method based on measuring continuous eye color variations using high-resolution digital full-eye photographs (28). Andersen et al (29) developed a Digital Iris Analysis Tool, which can be used to automatically identify and extract irises from high resolution digital images as well as calculate the so called Pixel Index of the Eye describing the eye color quantitatively. Such a detailed approach should reduce potential problems with correct categorization of eye colors. After all, further studies regarding additional genes and polymorphisms, as well as their interactions, contributing to variation in iris colors, especially non-blue and non-brown colors, should have a bearing on the improvement of their prediction accuracy (8,14,22,27). Multiple genes and their interactions are involved in the development of eye color variation, so further investigations of new pigmentation genes and SNP markers should be essential for more precise prediction of eye color, especially in the intermediate domain. We confirmed here that the model based prediction of eye color from DNA data was a reliable tool that can be useful in forensic investigations. Prediction of eye color from DNA extracted from biological traces may be used in criminal cases with unknown suspects. This tool may also supplement anthropological investigations by providing information about the eye color of a suspect. However, it is worth noting that forensic DNA phenotyping should be performed with care since it provides evidence that is associated with a relatively high error rate, particularly in case of some phenotypic categories like intermediate eye color.

In conclusion, our study contributed to the body of evidence on eye color phenotype variation across Europe, as well as on genotype distribution in the six eye color informative IrisPlex SNPs, providing data for Slovenian population sample. The obtained results confirmed the utility of the IrisPlex prediction model for accurate prediction of blue and brown eye colors. Further studies are needed to explain the remaining variation in human eye color and open up possibility for prediction of a complete spectrum of eye colors in humans.

## Additional Material

Supplementary Tables 1 na2
