# Supplementary material for: Prediction of eye color in the Slovenian population using the IrisPlex SNPs
Source: Croat Med J. 2013 Aug;54(4):381–6. doi: 10.3325/cmj.2013.54.381 (PMC3760663; doi:10.3325/cmj.2013.54.381)
Supplement: Supplementary Tables 1 na2 [file CroatMedJ_54_s001.pdf]

Supplementary tables 1 and 2

Supplementary Table 1: PCR primer sequences of the six IrisPlex polymorphisms including the two analyzed for the purpose of this study (SNPs rs12896399 and rs12203592)

**SNP-ID**	**gene**	**forward primer**	**reversed primer**	**ref.**
**rs12913832**	HERC2	TGATAGCGTGCAGAACTTGACA	TAATTCAAAATGCCCCCAAG	Eiberg, 2008
**rs1393350**	TYR	GGTGAATGATAACACGAACAGATT	GATGCGTGCATATCCACCAACT	Sulem, 2007
**rs1800407**	OCA2	TGAAAGGCTGCCTCTGTTCT	TATGGTCACAGGCGTGAAGA	Duffy, 2007
**rs16891982**	*SLC45A2*	GGAAAACACGGAGTTGATGC	CCAGAGGTGGAGAAGCAGAG	Soejima,2007
**rs12896399**	*SLC24A4*	CTGGCGATCCAATTCTTTGT	GACCCTGTGTGAGACCCAGT	Walsh, 2013
**rs12203592**	*IRF4*	AGGGCAGCTGATCTCTTCAG	GCTTCGTCATATGGCTAAACCT	Walsh, 2013

Supplementary Table 2: SBE primer sequences of the six IrisPlex polymorphisms including the two analyzed for the purpose of this study (SNPs rs12896399 and rs12203592)

**SNP-ID**	**gene**	**SBE primer length (bp)**	**SBE extension primer (5'-3')**	**primer conc. (μM)**	**alleles detected**	**ref.**
**rs12913832**	HERC2	25	TGATGATAGCGTGCAGAACTTGACA	1,50	A/G	Eiberg, 2008
**rs1393350**	TYR	50	GACTGACTGACTGACTGACTGACTGACTGAGTAAAAGACCACACAGATTT	1,00	A/G	self - designed
**rs1800407**	OCA2	55	GACTGACTGACTGACTGACTGACTGACTGACGTGGCCAGGCATACCGGCTCTCCC	0,01	A/G	Duffy, 2007
**rs16891982**	*SLC45A2*	45	GACTGACTGACTGACTAGAGGAGTCGAGGTTGGATGTTGGGGCTT	0,65	C/G	Soejima, 2007
**rs12896399**	*SLC24A4*	53	tttttttttttttttttttttttttttttttttttttttttttttttttttttttttttttttttTCTTTAGGTCAGTATATTTTGGG	0,15	G/T	Walsh, 2013
**rs12203592**	*IRF4*	35	tttttttttttttttttttttttttttttttttttttttttttttTCCACTTTGGTGGGTAAAAGAAGG	0,3	C/T	Walsh, 2013
