# Supplementary material for: Prediction of eye color in the Slovenian population using the IrisPlex SNPs
Source: Croat Med J. 2013 Aug;54(4):381–6. doi: 10.3325/cmj.2013.54.381 (PMC3760663; doi:10.3325/cmj.2013.54.381)
Supplement: Supplementary Table 3 [file CroatMedJ_54_s002.pdf]

Supplementary Table 3: Genotypes for the six currently most eye colour-informative SNPs and eye color prediction probabilities for 105 Slovene volunteers.

Identification number	Population	Volunteers eye color	rs16891982	rs12203592	rs1800407	rs12913832	rs12896399	rs1393350	blue (p)	intermediate (p)	brown (p)
1	Slovenenian	blue	G	C	GA	C	GT	C	0,945	0,045	0,011
2	Slovenenian	blue	G	C	G	C	GT	C	0,919	0,048	0,033
3	Slovenenian	intermediate	G	C	GA	CT	T	C	0,202	0,286	0,512
4	Slovenenian	blue	G	C	G	C	GT	CT	0,937	0,041	0,022
5	Slovenenian	brown	G	C	GA	T	G	C	0,008	0,071	0,921
6	Slovenenian	blue	G	C	GA	CT	G	C	0,419	0,211	0,371
7	Slovenenian	blue	G	C	G	C	T	C	0,870	0,076	0,053
8	Slovenenian	intermediate	G	C	G	CT	GT	C	0,150	0,140	0,711
9	Slovenenian	brown	G	C	GA	T	GT	C	0,005	0,071	0,924
10	Slovenenian	blue	G	C	GA	C	GT	CT	0,955	0,038	0,007
11	Slovenenian	brown	G	C	G	CT	T	C	0,094	0,148	0,758
12	Slovenenian	brown	G	C	G	CT	G	C	0,229	0,128	0,643
13	Slovenenian	brown	G	C	GA	T	T	C	0,003	0,071	0,926
14	Slovenenian	brown	G	C	G	CT	GT	CT	0,207	0,161	0,632
15	Slovenenian	intermediate	GC	C	GA	CT	GT	CT	0,143	0,222	0,635
16	Slovenenian	blue	G	C	G	C	G	CT	0,962	0,025	0,013
17	Slovenenian	intermediate	G	CT	G	CT	GT	CT	0,283	0,241	0,476
18	Slovenenian	intermediate	G	C	G	CT	G	C	0,229	0,128	0,643
19	Slovenenian	intermediate	G	C	G	C	G	C	0,950	0,030	0,020
20	Slovenenian	brown	G	C	G	CT	T	CT	0,134	0,175	0,691
21	Slovenenian	blue	G	CT	G	C	G	CT	0,965	0,027	0,007
22	Slovenenian	brown	G	C	GA	T	G	C	0,008	0,071	0,921
23	Slovenenian	intermediate	G	C	G	CT	G	T	0,393	0,152	0,455
24	Slovenenian	intermediate	G	CT	GA	T	T	CT	0,007	0,163	0,830
25	Slovenenian	brown	G	CT	G	CT	G	C	0,317	0,193	0,490
26	Slovenenian	blue	G	C	G	C	GT	C	0,919	0,048	0,033
27	Slovenenian	blue	G	C	G	C	GT	C	0,919	0,048	0,033
28	Slovenenian	blue	G	C	G	C	G	CT	0,962	0,025	0,013
29	Slovenenian	brown	G	C	G	CT	GT	T	0,277	0,179	0,543
30	Slovenenian	brown	G	C	GA	CT	GT	C	0,299	0,253	0,448
31	Slovenenian	blue	G	C	G	C	T	CT	0,899	0,066	0,035
32	Slovenenian	blue	G	C	G	C	G	T	0,970	0,021	0,009
33	Slovenenian	blue	G	C	G	C	T	C	0,870	0,076	0,053
34	Slovenenian	blue	G	C	G	C	GT	C	0,919	0,048	0,033
35	Slovenenian	blue	G	C	GA	C	G	CT	0,973	0,023	0,004
36	Slovenenian	blue	G	C	G	C	GT	CT	0,937	0,041	0,022
37	Slovenenian	brown	GC	CT	GA	T	G	CT	0,005	0,087	0,908
38	Slovenenian	blue	G	C	G	C	G	C	0,950	0,030	0,020
39	Slovenenian	brown	G	C	G	CT	GT	C	0,150	0,140	0,711
40	Slovenenian	blue	G	C	G	C	GT	CT	0,937	0,041	0,022
41	Slovenenian	blue	G	C	G	C	GT	C	0,919	0,048	0,033
42	Slovenenian	blue	G	C	G	C	GT	CT	0,937	0,041	0,022
43	Slovenenian	blue	G	C	G	C	T	C	0,870	0,076	0,053
44	Slovenenian	intermediate	G	C	G	C	GT	C	0,919	0,048	0,033
45	Slovenenian	blue	G	C	G	C	GT	C	0,919	0,048	0,033
46	Slovenenian	brown	G	C	G	CT	T	C	0,094	0,148	0,758
47	Slovenenian	blue	G	C	GA	C	G	C	0,966	0,027	0,007
48	Slovenenian	intermediate	G	C	GA	T	G	CT	0,012	0,090	0,898
49	Slovenenian	blue	G	C	G	C	GT	C	0,919	0,048	0,033
50	Slovenenian	brown	G	CT	G	CT	GT	C	0,215	0,221	0,564
51	Slovenenian	blue	G	C	G	C	T	C	0,870	0,076	0,053

52	Slovenenian			brown	GC	C	G	CT	GT	C	0,040	0,083	0,877
53	Slovenenian		intermediate		G	CT	G	CT	G	C	0,317	0,193	0,490
54	Slovenenian		intermediate		G	C	GA	CT	GT	CT	0,375	0,264	0,361
55	Slovenenian	blue			GC	C	G	C	G	C	0,857	0,059	0,085
56	Slovenenian			brown	G	C	G	T	G	C	0,003	0,026	0,971
57	Slovenenian		intermediate		G	CT	G	CT	GT	T	0,359	0,254	0,387
58	Slovenenian		intermediate		G	CT	G	CT	GT	C	0,215	0,221	0,564
59	Slovenenian			brown	G	C	G	CT	GT	CT	0,207	0,161	0,632
60	Slovenenian	blue			G	C	G	C	GT	CT	0,937	0,041	0,022
61	Slovenenian	blue			G	C	G	C	G	CT	0,962	0,025	0,013
62	Slovenenian	blue			G	CT	G	C	G	CT	0,965	0,027	0,007
63	Slovenenian			brown	G	C	G	T	G	C	0,003	0,026	0,971
64	Slovenenian		intermediate		GC	C	GA	CT	GT	CT	0,143	0,222	0,635
65	Slovenenian	blue			G	C	G	C	G	C	0,950	0,030	0,020
66	Slovenenian			brown	G	C	G	CT	GT	C	0,150	0,140	0,711
67	Slovenenian		intermediate		G	C	GA	CT	GT	C	0,299	0,253	0,448
68	Slovenenian	blue			G	C	G	C	T	CT	0,899	0,066	0,035
69	Slovenenian	blue			G	C	GA	CT	G	CT	0,503	0,211	0,286
70	Slovenenian		intermediate		GC	C	G	CT	GT	C	0,040	0,083	0,877
71	Slovenenian			brown	G	C	G	CT	T	CT	0,134	0,175	0,691
72	Slovenenian			brown	G	C	G	T	G	C	0,003	0,026	0,971
73	Slovenenian			brown	G	C	G	CT	T	C	0,094	0,148	0,758
74	Slovenenian		intermediate		G	C	G	CT	GT	C	0,150	0,140	0,711
75	Slovenenian	blue			G	CT	G	C	T	C	0,885	0,085	0,030
76	Slovenenian	blue			G	C	G	C	G	C	0,950	0,030	0,020
77	Slovenenian	blue			G	C	G	C	GT	CT	0,937	0,041	0,022
78	Slovenenian		intermediate		G	CT	G	CT	G	C	0,317	0,193	0,490
79	Slovenenian		intermediate		G	C	GA	CT	G	C	0,419	0,211	0,371
80	Slovenenian		intermediate		G	C	G	CT	G	C	0,229	0,128	0,643
81	Slovenenian	blue			G	C	G	C	GT	T	0,951	0,034	0,014
82	Slovenenian			brown	G	C	G	T	GT	C	0,002	0,026	0,972
83	Slovenenian		intermediate		G	CT	G	CT	G	CT	0,400	0,203	0,397
84	Slovenenian		intermediate		G	C	G	CT	T	CT	0,134	0,175	0,691
85	Slovenenian		intermediate		G	C	G	C	GT	C	0,919	0,048	0,033
86	Slovenenian			brown	G	C	GA	CT	G	C	0,419	0,211	0,371
87	Slovenenian		intermediate		G	C	G	C	GT	C	0,919	0,048	0,033
88	Slovenenian	blue			G	C	GA	C	GT	CT	0,955	0,038	0,007
89	Slovenenian	blue			G	C	G	C	GT	C	0,919	0,048	0,033
90	Slovenenian	blue			G	C	G	C	G	C	0,950	0,030	0,020
91	Slovenenian			brown	G	C	G	CT	GT	C	0,150	0,140	0,711
92	Slovenenian	blue			G	C	G	C	G	CT	0,962	0,025	0,013
93	Slovenenian			brown	G	C	GA	T	G	CT	0,012	0,090	0,898
94	Slovenenian		intermediate		G	C	G	CT	GT	CT	0,207	0,161	0,632
95	Slovenenian			brown	GC	C	G	CT	GT	C	0,040	0,083	0,877
96	Slovenenian	blue			G	C	G	C	T	C	0,870	0,076	0,053
97	Slovenenian	blue			G	C	G	C	GT	CT	0,937	0,041	0,022
98	Slovenenian	blue			G	C	G	C	T	T	0,921	0,056	0,023
99	Slovenenian		intermediate		G	CT	G	T	GT	C	0,003	0,051	0,947
100	Slovenenian			brown	G	C	A	T	GT	C	0,013	0,178	0,809
101	Slovenenian	blue			G	C	G	C	G	C	0,950	0,030	0,020
102	Slovenenian			brown	G	CT	G	CT	G	C	0,317	0,193	0,490
103	Slovenenian	blue			G	C	G	C	G	T	0,970	0,021	0,009
104	Slovenenian			brown	G	C	G	T	GT	CT	0,002	0,034	0,964
105	Slovenenian	blue			G	C	G	C	GT	CT	0,937	0,041	0,022
