# Supplementary material for: Prediction of eye color in the Slovenian population using the IrisPlex SNPs
Source: Croat Med J. 2013 Aug;54(4):381–6. doi: 10.3325/cmj.2013.54.381 (PMC3760663; doi:10.3325/cmj.2013.54.381)

Supplementary figure. Eye color images of nine Slovenian volunteers and their estimated IrisPlex eye color prediction probabilities for blue (Bl), intermediate (Int), and brown (Br) color. Images are ordered based on phenotype information.

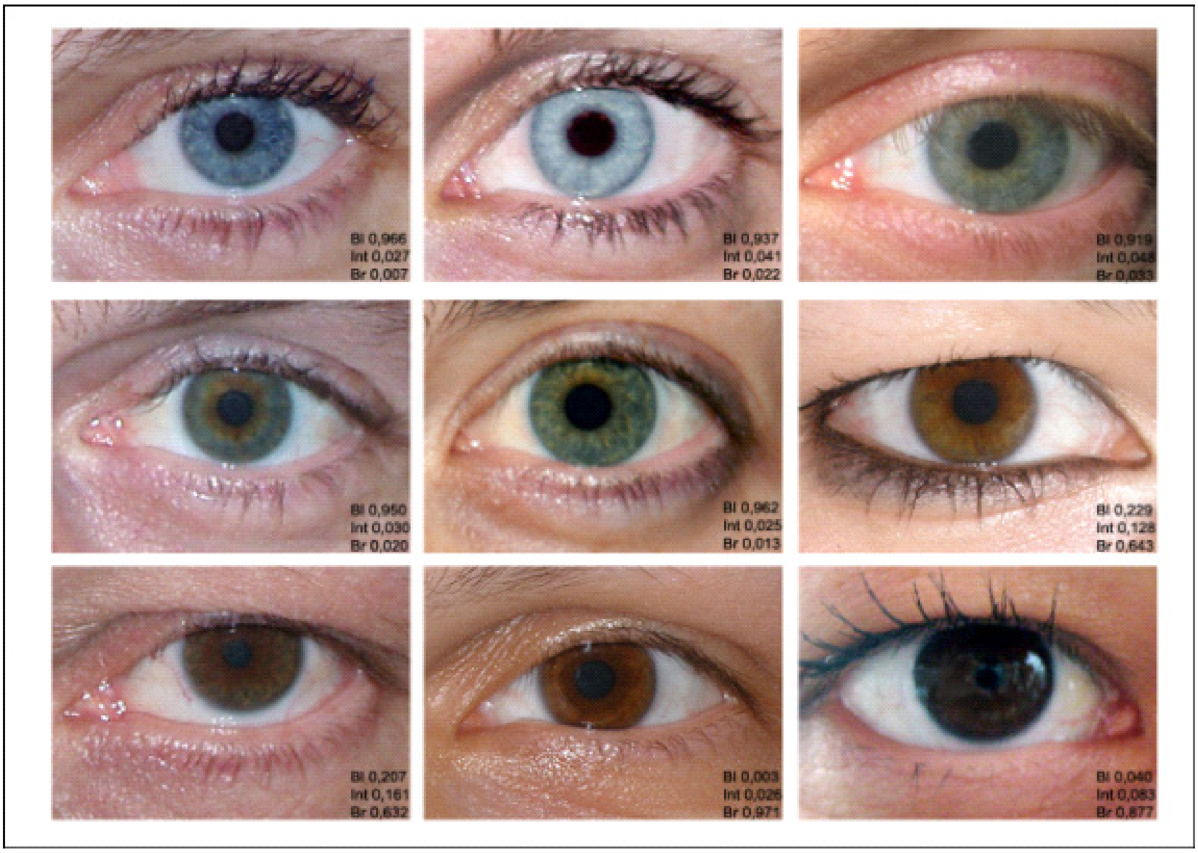

Supplement: Supplementary Figure [file CroatMedJ_54_s003.pdf]
